# Some like it hot: Variable impact of a tailpiece heating device on different gram-negative bacteria

**DOI:** 10.1017/ash.2023.318

**Published:** 2023-09-29

**Authors:** Stacy Park, Shireen Kotay, Katie Barry, Joanne Carroll, April Attai, William Guilford, Amy Mathers

## Abstract

**Background:** Transmission of multidrug-resistant bacteria to patients from colonized hospital sink drains has prompted attempts to interrupt transmission through a variety of interventions directed at the wastewater environment. We previously found that use of a heating device designed to disrupt biofilm formation between the P trap and the sink drain, which is the major point of dispersal of bacteria to the patient-care environment, was associated with reduced risk of detectable gram-negative organisms on hospital sink drains. However, there was no observed effect on some important pathogens, including *Pseudomonas aeruginosa* and *Stenotrophomonas maltophilia*. We hypothesized that heating to a higher temperature would provide additional efficacy in preventing drain colonization. **Methods:** As part of a previous randomized study, 54 tailpiece heaters were installed in 3 intensive care units in an academic hospital and 2 acute-care units in an associated regional hospital; half of these devices were shams (ie, no heat). The devices were programmed to heat for 1 hour every fourth hour. Prior to this study, a device update increased the heating temperature (during the previous study the median heated temperature was 65.9°C). Sink drains and P traps were sampled monthly. Samples were assessed for semiquantitative growth of gram-negative bacteria on MacConkey agar, looking especially for *P. aeruginosa* and *S. maltophilia*. Frontline personnel were blinded to device assignment. **Results:** The mean heated temperature reached was 74.4°C. Based on proportional odds logistic regression (wherein the odds ratio reflects the likelihood of a given sample falling in a lower microbiologic burden level versus the levels above it), the heating device was associated with increased likelihood of lower microbiologic burden at the drain level for general growth on MacConkey agar (OR, 2.47; 95% CI, 1.11–5.51) and for growth of *S. maltophilia* (OR, 5.39; 95% CI, 2.20–13.18). The device did not have an effect on burden of Enterobacterales (OR, 1.38; 95% CI, 0.58–3.24). For *P. aeruginosa*, there was a trend toward decreased likelihood of lower microbiologic burden (OR, 0.41; 95% CI, 0.18–1.07) that did not reach statistical significance at the drain level, and the heating device was associated with decreased likelihood of lower microbiologic burden of *P. aeruginosa* at the P-trap level (OR, 0.20; 95% CI, 0.10–0.39). **Conclusions:** Heat disruption of biofilm between the P trap and sink may be a promising strategy for prevention of hospital sink drain colonization; however, the impact is variable across different bacterial species. Further understanding of the dynamics of the microbiome within wastewater is needed.

**Figure:**
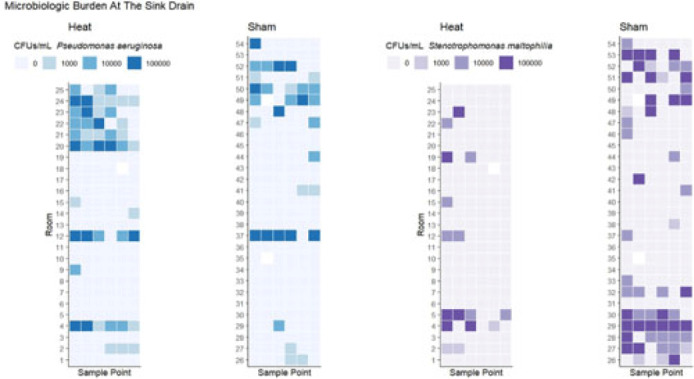

**Disclosures:** None

